# The Influence of Foreign Direct Investment on Physical Health of Rural-Urban Migrants—Empirical Evidence from China Migrants Dynamic Survey

**DOI:** 10.3390/ijerph20054268

**Published:** 2023-02-28

**Authors:** Guixin Han, Pengcheng Liu, Yihang Zhao, Yinyin Liang, Xiaojie Wang

**Affiliations:** 1 School of Management, Shandong University, Jinan 250100, China; 2 School of Economics, Qingdao University, Qingdao 266100, China; 3 School of Management, Ocean University of China, Qingdao 266100, China; 4 Nottingham University Business School China, University of Nottingham Ningbo China, Ningbo 315100, China

**Keywords:** foreign direct investment, physical health, rural-urban migrants in China, employment rights

## Abstract

The purpose of this study is to explore the influence of Foreign Direct Investment (FDI) on rural-urban migrants’ physical health and its influencing mechanism. A total of 134,920 rural-urban migrant samples are matched based on the China Migrants Dynamic Survey in 2017 and the China Urban Statistical Yearbook in 2016. On the basis of the samples, a Binary Probit Model is used to explore the relationship between the degree of FDI and rural-urban migrants’ physical health. The results show that compared with migrants who lived in cities with a lower FDI level, rural-urban migrants who lived in cities with a higher FDI level are better in physical health. The results of the mediation effect model show that the degree of FDI has a significant positive impact on employment rights and benefits the protection of rural-urban migrants, improving rural-urban migrants’ physical health, which means employment rights and benefits protection plays an intermediary role in the process of FDI affecting rural-urban migrants’ physical health. Therefore, when formulating public policies such as plans to improve the physical health of rural-urban migrants, not only the availability of medical services for rural-urban migrants needs to be improved, but the positive spillover effect of FDI should be taken into account. By doing so, FDI can positively affect the physical health of rural-urban migrants.

## 1. Introduction

In China, due to the opening-up policy, especially China’s accession to World Trade Organization (WTO), the high employment demand and wage levels in urban areas have attracted a large amount of rural surplus labor, which has led to a rapid increase in the number of rural-urban migrants. According to the National Census Statistics in 2020, China has a 376 million floating population, accounting for 26.63% of the total population. Due to the restrictions of China’s unique household registration policy, it is difficult for rural-urban migrants to enjoy equal employment treatment with urban residents. Furthermore, the lack of employment rights, imperfect medical security, and poor living and working environments make rural-urban migrants face higher physical health risks. Hence, physical health management of rural-urban migrants has also become an urgent social and public health problem. The influence of external environmental factors on rural-urban migrants’ physical health has received extensive attention [1,2]. Scholars have conducted in-depth discussions on factors such as urban air quality [3] and city size [4]. Although Foreign Direct Investment (FDI) is an important factor causing rural-urban migrants to flow, its impact on rural-urban migrants’ physical health has not received enough attention.

Studies on the impact of FDI on rural-urban migrants are common, such as the research on the relationship between FDI and the jobs of rural-urban migrants [5], and the research on the relationship between FDI and the social network of rural-urban migrants [6]. However, the existing literature does not pay enough attention to the internal relationship between FDI and individual health. Moreover, the intersection of literature from different disciplines (such as international trade and health economics) constantly implies the necessity and practical significance of our research topic. In the research on the relationship between FDI and individual health, scholars mainly focus on the health of adults [7,8] and infants [9] in the host country, while ignoring the impact of FDI on rural-urban migrants’ physical health. Some scholars have noticed that FDI will lead to the migration of rural labor into cities, causing the minor children of rural-urban migrants to become left-behind children [10], which in turn will exert a negative influence on the physical health of the children [11]. However, they have not further explored the impact of FDI on this specific group’s physical health, which is exactly the research content of this paper.

Theoretically, the relationship between FDI and rural-urban migrants’ physical health is uncertain. On the one hand, the literature based on the data of the China Migrants Dynamic Survey (CMDS) showed that trade liberalization would significantly increase Migrants’ wages [12]. In addition, more foreign direct investment means better working conditions and better welfare [13,14]. These are beneficial to rural-urban migrants’ physical health [15]. On the other hand, more foreign direct investment in cities will also result in longer working hours, consumption of tobacco or unhealthy foods, and rising levels of harmful pollution, all of which directly harm physical health [16,17,18]. However, these are harmful to rural-urban migrants’ physical health. Therefore, it is necessary to conduct an empirical analysis based on the data of migrants in China to determine which impact of FDI on migrants’ physical is more prominent. This is also an important part of this paper.

Most of the research on the impact mechanism of FDI participation and rural-urban migrants’ physical health use income as an intermediary variable [19,20]. However, on the basis of China’s social condition, it is more practical to use employment rights and benefits protection as an intermediary when analyzing FDI participation’s effects on rural-urban migrants’ physical health. The reasons are as follows: Firstly, most rural-urban migrants in China have low health management ability and lack health knowledge [21,22]. Therefore, part of the income of rural-urban migrants is used for their daily expenses. The rest is mainly used to improve the living standards of other members (such as a spouse, children, or parents) in rural-urban migrant families [23] or improve children’s educational conditions [24], rather than invest in their own health. Therefore, according to the actual situation of rural-urban migrants, the relationship between income level and their health status needs to be further confirmed. Secondly, according to the Grossman model of health needs [25], health is an investment. Furthermore, employment rights and benefits protection provides an important guarantee for the disease treatment and health recovery of rural-urban migrants and is conducive to rural-urban migrants obtaining resources for health investment. Therefore, employment rights and benefits protection is an important variable affecting rural-urban migrants’ physical health. However, in China, rural-urban migrants generally have a relatively low level of education (according to CMDS2017 data, 59.68% of rural-urban migrants have a junior high school education level or below). Coupled with the restriction of the household registration system, most rural-urban migrants are faced with the problem of heavy work but lack of employment rights and benefits [26]. FDI participation will have an impact on this situation. Specifically speaking, foreign-funded enterprises not only have a clearer awareness of their social responsibilities but also have important effects on demonstration and competition on host country enterprises [27], thus enhancing employment rights and benefits protection in the region where they locate. Many multinational companies not only take the lead in strictly implementing corporate social responsibility in China, but also invest a lot of material and human resources to organize and participate in relevant conferences and activities in China, and actively introduce and promote the concept of corporate social responsibility of multinational companies. For example, as early as 2006, many foreign-funded enterprises jointly issued the Beijing Declaration on Social Responsibility in Beijing. The declaration promises that all foreign-funded enterprises participating in the conference would actively fulfill their social responsibilities in 12 aspects, including law, tax payment, intellectual property, employment rights and benefits [27]. Therefore, by introducing the advanced concept of social responsibility to China, transnational enterprises perform as the demonstrator and booster of the social responsibility campaign among Chinese enterprises, which is beneficial to increase the possibility of the improvement of employment rights and benefits in domestic enterprises [28]. Furthermore, it can be speculated that FDI participation will have a positive impact on rural-urban migrants’ physical health by improving employment rights and benefits protection. However, few literature studies have carried out rigorous empirical research on this. Assessing the impact of FDI participation on rural-urban migrants’ physical health is an important aspect of studying rural-urban migrants and the impact of FDI participation on the host country’s labor market, which has important practical significance.

Combined with the above analysis, this paper systematically examines the impact of FDI on rural-urban migrants’ physical health based on the data from the China Migrants Dynamic Survey in 2017 (CMDS2017). The marginal contribution is mainly reflected in the following two points: (1) The rural-urban migrants have certain particularities. This paper takes the rural-urban migrants in China as the research object and discusses the impact of FDI on rural-urban migrants’ physical health. This provides additional information for a comprehensive review and assessment of the welfare effects and implicit costs of “globalization”. (2) From the perspective of the realistic situation of rural-urban migrants in China, this paper explains the intermediary role of employment rights and benefits protection. It reveals the path that FDI affects rural-urban migrants’ physical health and enriches the relevant research.

## 2. Materials and Methods

### 2.1. Data Source

The data were derived from the China Urban Statistical Yearbook in 2016 and the China Migrants Dynamic Survey in 2017 (CMDS2017). The China Urban Statistical Yearbook is an informative annual report of the social-economic development status of Chinese cities released by the National Bureau of Statistics of the People’s Republic of China. It includes data on the population, finance, foreign trade, economic development, resources, and environment of 656 cities in China (including prefecture-level and above cities and county-level cities). The China Migrants Dynamic Survey CMDS is an annual large-scale national migrant population sampling survey initiated by the National Health Commission of the People’s Republic of China. The stratified, multistage, and proportional PPS method is adopted. The respondents consisted of those who were aged over 15, had lived in the destination for more than one month, and had a household registration beyond the local county or city. CMDS2017 covers 31 provinces (autonomous regions and municipalities) in mainland China. The survey covers the basic information of respondents and their family members (such as gender, age, education level, etc.), the scope and time of migration, employment and social security, medical security, basic public health services, and other modules, and the questionnaire has good authenticity and reliability.

It should be noted that the effect of FDI on the physical health of individuals often has a lag. Therefore, the explanatory variables and city-level control variables use data with a lag of one period in this paper. We match the China Urban Statistical Yearbook in 2016 with CMDS2017 according to the indicator of migrants’ inflow cities. After excluding incomplete answers and invalid data samples, we finally obtained 134,920 valid samples distributed in 256 cities.

### 2.2. The Basic Model

In this paper, the dependent variable is rural-urban migrants’ physical health, which is a binary dummy variable, so a binary Probit model is set as follows:

$$Health{\left(1,0\right)}_{ij}=\beta_{1}+cFDI_{ij}+\gamma_{1}X_{i}+\varepsilon_{ij}$$

where $Health{\left(1,0\right)}_{ij}$ denotes the physical health of immigrant *i* in city *j*; $FDI_{ij}$ represents the participation of foreign investment in city 𝑗 where immigrant *i* flows into. The larger the value, the deeper the degree of foreign direct investment; $X_{i}$ is a set of control variables, and $\varepsilon_{ij}$ is the error term.

### 2.3. Selection of Variables

#### 2.3.1. Dependent Variable

This article used the individual’s illness or physical condition as an indicator for the measurement of the explained variable (physical health). The survey item on this variable in the CMDS2017 data was: “Have you been sick (injured) or unwell in the last year?” with response categories of “Yes, the last time happened within two weeks”, “Yes, the last time was two weeks ago” and “no”. Individuals who were reported to be “Yes, the last time happened within two weeks”, “Yes, the last time was two weeks ago” were classified as those who had bad physical condition (=0). Individuals who were reported to be “no” were classified as those who were in good physical condition (=1).

#### 2.3.2. Independent Variables

The independent variable is the degree of FDI, which is measured by the proportion of city’s FDI that is actually used for its GDP. The city’s FDI that is actually used (unit: ten thousand US dollars) is converted according to the 2016 exchange rate, to ensure that the city’s foreign capital that actually used and GDP are measured in the same currency unit.

#### 2.3.3. Mediating Variable

The intermediary variable is the protection of employment rights and benefits. Employment rights and benefits include the right to occupational safety and health protection, the right to enjoy social insurance and welfare (such as medical insurance, industrial injury insurance, etc.), the right to rest and leisure, etc. The protection of employment rights and benefits is measured by whether the rural-urban migrants had signed labor contracts with employers. The survey item on this variable in the CMDS2017 data was: “What kind of labor contract do you sign with your current work unit (employer)?” Based on the respondents’ answers, the intermediary variable was measured with a dummy variable (1 for “sign an employment contract” and 0 for “no labor contract”).

#### 2.3.4. Control Variables

The control variables involved in this study are grouped into two categories. The first category elicited sociodemographic characteristics of the participants: age referred to the actual age of the interviewee; gender was measured with a dummy variable (1 for males and 0 for females); educational levels were expressed as “middle school and below (=1)”, “high school/vocational school (=2)”, and “college and above (=3)”. Mobility duration was the time of participants migrating to the city. The household income level was measured by the question: “What was the average monthly total income of your household in the past year”, and logarithmic processing was performed. The second category assessed the characteristics of the city where the participants flowed into the following categories: the industrial structure refers to the proportion of the total output value of the city’s tertiary industry in the city’s Gross Regional Product; the proportion of fiscal revenue is the ratio of public fiscal revenue to GDP; the proportion of fiscal expenditure is the ratio of public fiscal expenditure to GDP.

### 2.4. Mediating Effect Model

To verify the mechanism that the degree of FDI affects rural-urban migrants’ physical health, this paper uses the Sobel test [29] to test the mediating effect. In this paper, we symbolically note the direct effect of the independent variable on the dependent variable as “FDI → Physical Health” and the indirect effect of the independent variable on the dependent variable through the mediator M as “FDI → M → Physical Health”. First, build the following regression model:

(1) $$Health_{ij}=\beta_{1}+cFDI_{ij}+\gamma_{1}X_{i}+\varepsilon_{ij}$$

(2) $$M=\beta_{2}+aFDI_{ij}+\gamma_{2}X_{i}+\varepsilon_{ij}$$

(3) $$Health_{ij}=\beta_{3}+c^{\prime }FDI_{ij}+bM+\gamma_{3}X_{i}+\varepsilon_{ij}$$

where $Health_{ij}$ represents the dependent variable, $FDI_{ij}$ represents the independent variable, M is the mediator variable, $X_{i}$ is a set of control variables, and $\varepsilon_{ij}$ denotes the error term.

The mediation effect test is as follows: (I) Calculate the Sobel test statistic $Z_{\mathrm{Sobel}}=ab/\sqrt{a^{2}s_{b}^{2}+b^{2}s_{a}^{2}}\;$. The significance of the parameter estimate c in Equation (1) indicates whether there exists a direct impact of the independent variable on the dependent variable (FDI→ Physical Health), and Equations (2) and (3) are fit to determine whether there exists an indirect effect of independent variable on dependent variable through the mediator M (FDI→ M → Physical Health). To determine whether there is a significant mediation effect, we can extract estimate a and its standard error $S_{a}$ from Equation (2) and extract the estimate b and $S_{b}$ from Equation (3). Mediation can be tested via a z-test. (II) Since $Z_{Mediation}$ is a normal distribution, we can test the significance of the mediating effect. At the 0.05 significance level, if $\left|Z_{Mediation}\right|$ > 1.96, then the intermediate path is proved to be present and significant.

### 2.5. Endogenous Problems

The econometric model in this article may also have endogenous problems. Unobservable variables, such as differences in individual physical fitness, may affect physical health, and such variables are not included in the model, which may confound the main relationship between variables. Another problem that needs to be solved is two-way causality. The physical health of workers in the host country affects FDI inflows [30], and this may lead to bidirectional causality issues. In this paper, the inverse of foreign market distance is used as the instrumental variable to deal with the endogenous problem. In this study, the foreign market refers to the market formed in other countries outside China. It is a market formed on the basis of China’s foreign trade. The instrumental variable (the reciprocal of foreign market distance) is measured by the inverse of the distance from each city to the coastline. Therefore, the value of the instrumental variable is positively correlated with FDI. The reasons for selecting this variable as the instrumental variable are as follows: The foreign market distance has a strong correlation with FDI [31]. However, no evidence shows that foreign market distance is related to an interviewee’s physical health. Because migrants generally have a relatively low level of education, there are many obstacles to getting better medical resources abroad. Although the migration of migrants between provinces is relatively convenient in China, there is a big difference between domestic migration and transnational migration. In addition, the level of migrants’ income is generally low, and they cannot afford better health care abroad. Moreover, no research has shown that the distance from the city to the coastline is related to the physical health of the residents in the city. Therefore, foreign market distance is a more suitable instrumental variable.

### 2.6. Statistical Analysis

A descriptive analysis using mean, standard deviation, frequencies, and percentages was computed to explain the samples and variables. A binary probit model was conducted to estimate the strength of the association between FDI and the indicator migrants’ physical health. In addition, variables for potential confounders (age, gender, education level, mobility duration, household income, industrial structure, the proportion of fiscal revenue, and the proportion of fiscal expenditure) were added to the models. We used a mediating effects model to identify the mediating effects of employment rights and benefits protection under the influence of FDI that affects migrants’ physical health. Statistical significance was defined as *p*-values < 0.05. Data analysis was conducted using the STATA 15.0 (Stata, College Station, TX, USA).

## 3. Results

### 3.1. Characteristics of Participants

Table 1 shows the demographic characteristics and outcome variables of the participants. Among 134,920 participants, the majority of respondents reported good physical condition (*n* = 68,455, 50.74%), and 49.26% of the respondents had been sick or unwell in the last year. The average age was 36.32 years old. In total, 68,905 (51.07%) were males and 66,015 (48.93%) were females. Generally, the respondents were not highly educated, with only 7.77% of migrants having a bachelor’s degree or above, and nearly 60% (*n* = 78,079, 57.87%) having a junior middle school education or below. The respondents’ average mobile duration was 7.13 years. The household income of the respondents was 7436.76 yuan (the logarithmic average was 8.71).

### 3.2. Characteristics of Cities Where the Migrants Flow Into

Table 1 also reports the characteristics of cities where the migrants flow into. The average FDI was 3%. The per capita GDP of the city was 82,448.22 yuan. The mean of the city’s industrial structure is 0.52, that is, the total output value of the tertiary industry accounts for 52% of the total output value of the city. The average ratio of public fiscal revenue to the city’s GDP is 0.11, and the average ratio of public fiscal expenditure to city’s GDP is 0.17.

### 3.3. Total Sample Regression Results

This article used binary probit model regression to investigate the influence of FDI on rural-urban migrants’ physical health. We cluster the errors at the district level. The benchmark regression results are shown in Table 2. Compared with migrants who lived in cities with lower FDI levels, rural-urban migrants who lived in cities with higher FDI levels had a higher probability of not experiencing “sickness or physical discomfort in the last year”, and it was statistically significant (coefficient = 5.686 ***, 95% CI: 3.557, 7.815). This showed that rural-urban migrants working in cities with higher FDI participation had a significantly greater probability of not having sick as compared to those who were working in cities with lower FDI participation, net of other controls. After calculating the marginal effect, we found that a standard deviation of FDI inflowing reduces the probability of rural-urban migrants’ “sickness or physical discomfort in the last years” by 4.44% [0.02 (standard deviation) × 2.222 (marginal effect)], accounting for 8.88% of the standard deviation of the rural-urban migrants “sickness or physical discomfort in the last years” (standard deviation for rural-urban migrants’ physical health is 0.500). That is, FDI can explain about 9% of rural-urban migrants’ “sickness or physical discomfort in the last years”. Expanding to the whole China level, the seventh national census showed that China had a 375.8 million floating population. Each increase of one standard deviation of FDI can prevent about 16.686 million migrants (375.8 million × 4.44%) from being troubled by “sickness or physical discomfort in the last year”. Therefore, the further increase in FDI brought about by the new round of high-level opening up will improve the physical condition and well-being of migrants.

### 3.4. Endogeneity Test

To solve the problem of endogeneity, this paper applied the two-stage IVProbit model for quantitative analysis. The results are shown in Table 3. It can be seen from the results of the first-stage test that the FDI was highly correlated with foreign market distance (Coefficient = 0.002 ***). The F statistic value far exceeded the threshold of the 10% bias level, which indicates that the instrumental variable is not a weak instrumental variable (T = 143.73, F = 7365.18, *p* = 0.000). The second-stage regression results showed that the FDI had a significant positive impact on rural-urban migrants’ physical health (Coefficient = 3.183 ***); this result is still robust after adding the control variables and is basically consistent with the binary probit model regression results. Therefore, after eliminating endogeneity, the results are robust and reliable.

### 3.5. Intermediary Effect Test

To analyze the mediating effects of employment rights and benefits protection, we used stepwise regression. First, we tested the influence of FDI on rural-urban migrants’ physical health, and the results are consistent with the results in Table 2; that is, the higher the degree of FDI, the better the physical condition of rural-urban migrants. Second, we examined the influence of FDI on the mediating variable (employment rights and benefits protection). Column (1) in Table 4 shows the results. The coefficient of the variable of FDI is significantly positive (Coefficient = 0.586 *), indicating that the higher the degree of FDI, the more likely it is to obtain the protection of employment rights and benefits. Third, we examined the influence of both the mediating variable and FDI on rural-urban migrants’ physical health. Column (2) shows that the coefficient of employment rights and benefits protection is significantly positive, indicating that there is a significant positive correlation between employment rights and benefits protection and rural-urban migrants’ physical health (Coefficient = 0.213 ***). Table 5 shows the statistical results of the mediation effect test. The absolute value of Z-test for variable employment rights and benefits protection is greater than 1.96, which indicates that the intermediate path is present and significant. That is, employment rights and benefits protection play an intermediary role in FDI influencing rural-urban migrants’ physical health. FDI has a demonstrative effect, helping to regulate the labor market in cities. This will help improve the probability of rural-urban migrants signing labor contracts with employers and the protection of their employment rights and benefits. In turn, improving rural-urban migrants’ physical condition.

## 4. Discussion

### 4.1. Implications

Based on the CMDS2017 data, this paper uses the binary Probit model to analyze the impact of FDI on rural-urban migrants’ physical condition. The results show that after controlling other variables that may affect rural-urban migrants’ physical health, the FDI has a significant positive impact on it. In other words, compared with migrants who lived in cities with a lower FDI level, rural-urban migrants who lived in those with a higher FDI level are more likely to have a better physical condition. China’s long-standing household registration system has limited more rural-urban migrants out of local resources, resulting in rural-urban migrants making great contributions to urban development, but not enjoying the same treatment as local residents. However, the entry of foreign-funded enterprises has brought higher standards of corporate social responsibility and has also intensified competition in the urban labor market. By improving employment rights and benefits protection in the region, it is possible for rural-urban migrants to enjoy medical resources and health care services in the city, thereby improving their physical health.

Specifically, foreign-funded enterprises have important demonstration and competition effects on local enterprises [32], both of which have an impact on employment rights and benefits protection of rural-urban migrants. From the perspective of the demonstration effect, the direct investment of multinational corporations in China is not limited to capital and technology. It also includes advanced concepts such as modern enterprise management mode, corporate social responsibility, and especially, employment rights and benefits protection [33]. Introducing the advanced concept of social responsibility to China, transnational enterprises perform as the demonstrator and booster of the social responsibility campaign among Chinese enterprises, which is beneficial to increase the possibility of the improvement of employment rights and benefits in domestic enterprises. From the perspective of the competition effect, FDI participation will have an impact on the labor market demand of the host country [34]. Therefore, when the labor supply is limited, the intensification of employment competition forces domestic enterprises to improve the rules and regulations related to employees’ employment rights and benefits protection. In addition, the increased demand for labor has improved the bargaining power of laborers, making it more likely that rural-urban migrants can obtain employment rights and benefits protection. Under the realistic background that urban public health services basically target the population with household registration, it is of great significance for rural-urban migrants to obtain employment rights and benefits protection. This means that rural-urban migrants can have rights such as medical insurance and work-related injury compensation, diminishing the barriers for rural-urban migrants to obtain medical care in cities [35] and having a positive impact on their physical health.

It is of great practical significance to improve the overall environment of employment rights and benefits protection in China. For domestic enterprises, it is necessary to enhance their own absorptive capacity [36] to maximize the positive external spillover effect of FDI, thus contributing to the improvement of the city’s overall employment rights and benefits protection [37].

In addition, the level of education and family income did not have a positive impact on the self-reported health status of migrants. The main reason for this phenomenon is that the overall health literacy of migrants is low [38], which makes the migrants lack attention to their health status. However, migrants with a higher education level and family income have higher health literacy [39] and pay more attention to health status. Simultaneously, they have more energy and money invested in improving their health status. Therefore, when encountering the same health problem (such as a disease with mild symptoms that does not affect the individual’s daily life, such as tolerable pain, cold, cough, etc.), migrants with a higher education level and family income are more likely to think that they are in an unhealthy status, which reduces the possibility of these individuals forming positive subjective health status. In addition, the duration of migration has a negative impact on rural-urban migrants’ physical health. The conclusions of this study support the healthy immigrant effect (HIE). The healthy immigrant effect (HIE) is the phenomenon in which immigrants show better health than their native-born counterparts but this healthier condition declines with the length of residence [40]. That is, rural-urban migrants’ physical health will gradually converge to the health level of local residents or even a lower level with the prolongation of residence [41,42]. According to the existing literature, this is mainly due to the long-term disadvantaged position of rural-urban migrants, who face more severe constraints when enjoying medical services and public basic services, resulting in accessibility barriers to medical resources [43]. In this paper, per capita GDP, the proportion of fiscal revenue, and the proportion of fiscal expenditure all have a significant negative impact on the self-reported health status of migrants. This is mainly related to the restriction and discrimination of the household registration system. Generally, cities with higher per capita GDP, a higher proportion of fiscal revenue, and fiscal expenditure are cities with a higher level of economic development, and the differentiation of resources between different groups is also more serious. As a vulnerable group, migrants are more likely to be excluded from local resources. The findings of Shao et al. [44] also showed that migrants in the more developed eastern regions of China used fewer public health services. In addition, there are obstacles for migrants to participate in social insurance in inflow cities and the high cost of migration also reduces the budget for migrants to obtain medical resources [45]. In this case, when migrants encounter minor health damages, they are more likely to choose to suffer by themselves instead of seeking medical treatment in time [46]. However, this approach will reduce the subjective assessment of migrants on their physical health, and they are more likely to feel that they are unhealthy. This also highlights the importance of employment rights and benefits protection for rural-urban migrants’ physical health.

### 4.2. Limitations

The limitations of this study are as follows: Firstly, although this paper uses an objective indicator (“sickness or physical discomfort in the last year”) to measure rural-urban migrants’ physical health, it is still derived from the self-reported data of rural-urban migrants, as a result of which, existing but unobserved physical health problems in rural-urban migrants cannot be completely ruled out. CMDS data does not cover more objective and detailed indicators related to physical health. Follow-up research can try more scientific measures of physical health variables. Secondly, this paper is a research paper based on cross-sectional data, focusing on the current physical condition of rural-urban migrants. If the data of tracking investigation into rural-urban migrants can be involved and analyzed through longitudinal study in future studies, it is possible to gain a more comprehensive understanding of the dynamic impact of FDI on rural-urban migrants’ physical health in terms of the process, influence and overall trend of impact. Thirdly, this paper examines the intermediary role of employment rights and benefits based on the reality of rural-urban migrants. However, under the current household registration system in China, the obtainment of employment rights and benefits protection is not equal to the same treatment as local residents, such as treatments such as accessibility to medical resources and health care services, which will affect rural-urban migrants’ physical health. Therefore, for future studies, contextual factors that are closely related to the realistic situation of rural-urban migrants can be added.

## 5. Conclusions

Rural-urban migrants account for a quarter of the total population in China. The exploration of rural-urban migrants’ physical condition and influencing factors is of great practical significance for the improvement of their physical health and the national physical level. Based on the China Migrants Dynamic Survey in 2017 (CMDS2017), this paper proves that FDI has a significant positive impact on rural-urban migrants’ physical health. The results of the intermediary effect show that FDI plays a role through the intermediary mechanism of employment rights and benefits protection.

According to the conclusion of this paper, the government should take the positive impact of FDI on rural-urban migrants’ physical health as an important consideration in evaluating the spillover effect of FDI and improving rural-urban migrants’ physical health. First, the government should pay attention to rural-urban migrants’ physical health and deepen the research on the factors affecting rural-urban migrants’ physical health by including FDI in the research scope, thus formulating targeted policies. Second, the government should optimize the business environment for attracting FDI, continue to deepen the introduction of FDI, and enhance the positive external spillover effect of foreign-funded enterprises to maximize the positive effect of FDI on rural-urban migrants’ physical health. Finally, the environment for employment rights and benefits protection in China can be improved as a whole by learning from the system of foreign-funded enterprises. For one thing, in the signing and performance of labor contracts by enterprises, the government should strengthen guidance and supervision, and earnestly safeguard the employment rights and interests of rural-urban migrants. For another, rural-urban migrants’ awareness of employment rights and benefits protection should be improved. The government should popularize the “Labor Contract Law” and the laws related to employment rights and benefits protection for rural-urban migrants and encourage them to sign labor contracts with enterprises before working.

## Figures and Tables

**Table 1:** Descriptive characteristics of participants (*n* = 134,920).

Variables	Categories	Mean *±* SD or *n* (%)
Physical Health, *n* (%)	Unhealthy	66,465 (49.26)
	Healthy	68,455 (50.74)
Age, mean (SD)		36.32 ± 9.43
Gender, *n* (%)	Male	68,905 (51.07)
	Female	66,015 (48.93)
Highest education level, *n* (%)	Middle school and below	78,079 (57.87)
	High school/vocational school	30,970 (22.95)
	College	15,389 (11.41)
	University and above	10,482 (7.77)
Mobility duration, mean (SD)		7.13 ± 5.89
Household income, mean (SD)		8.71 ± 0.68
The degree of FDI, mean (SD)		0.03 ± 0.02
Per capita GDP, mean (SD)		11.22 ± 0.47
Industrial structure, mean (SD)		0.52 ± 0.12
The proportion of fiscal revenue, mean (SD)		0.11 ± 0.04
The proportion of fiscal expenditure, mean (SD)		0.17 ± 0.06

**Table 2:** Binary probit model regression analysis of FDI influencing rural-urban migrants’ physical health.

Variables	Coefficient	Std. Err.	Z	95% CI
FDI	5.686 ***	1.086	5.24	(3.557, 7.815)
Age	0.010 ***	0.004	2.56	(0.002, 0.018)
Age × Age	−0.0001 *	0.000	−1.44	(−0.0002, 0)
Gender (ref: female)	0.064 ***	0.007	8.89	(0.050, 0.079)
Education level (reference: Middle school and below)				
High school/Vocational school	0.015	0.013	1.11	(−0.011, 0.040)
College	−0.030 *	0.019	−1.58	(−0.066, 0.007)
University and above	−0.043 **	0.030	−1.43	(−0.101, 0.016)
Mobility duration	−0.007 ***	0.001	−4.72	(−0.010, −0.004)
Household income	−0.071 ***	0.010	−7.00	(−0.091, −0.051)
Per capita GDP	−0.415 ***	0.073	−5.66	(−0.559, −0.272)
Industrial structure	1.499 ***	0.282	5.32	(−0.946, 2.052)
The proportion of fiscal revenue	−2.303 ***	1.021	−2.26	(−4.304, −0.301)
The proportion of fiscal expenditure	−0.240 *	0.585	−0.41	(−1.39, −0.906)
Sample size	134,920			

Note: *** Significant at *p* < 0.001. ** Significant at *p* < 0.01. * Significant at *p* < 0.05. CI, confidence interval.

**Table 3:** Endogeneity test of the influence of FDI on rural-urban migrants’ physical health.

	The First Stage (1)			The Second Stage (2)		
Variables	Coefficient	Std. Err.	95% CI	Coefficient	Std. Err.	95% CI
Foreign market distance	0.002 ***	0.000	(0.002, 0.002)			
FDI				3.183 ***	0.482	(2.238, 4.129)
Control variables	YES			YES		
Constant	−0.169	0.002	(−0.173, −0.164)	3.887 ***	0.193	(3.509, 4.264)
Sample size	134,920			134,920		
F-value of the first stage	7365.18					
Instrument variable T-value	143.73					
Wald test	31.30					
	(0.00)					

Note: *** Significant at *p* < 0.001. CI, confidence interval.

**Table 4:** Test of intermediary effect of FDI on rural-urban migrants’ physical health.

	Employment Rights and Benefits Protection (1)			Rural-Urban Migrants’ Physical Health (2)		
Variables	Coefficient	Std. Err.	95% CI	Coefficient	Std. Err.	95% CI
FDI	0.586 *	0.277	(0.0434, 1.128)	1.156	0.860	(−0.529, 2.840)
Employment rights and benefits protection				0.213 ***	0.034	(0.145, 0.280)
Control variables	YES			YES		
Constant	−4.120 ***	0.264	(−4.637, −3.604)	3.195 ***	0.824	(1.581, 4.809)
Sample size	68,792			68,792		

Note: *** Significant at *p* < 0.001. * Significant at *p* < 0.05. CI, confidence interval.

**Table 5:** The results of the mediation effect test.

Influence Path	Mediating Effect	$Z_{\mathrm{Sobel}}-\mathrm{Value}$	Result
FDI → Employment rights and benefits protection → physical health	0.125	2.003	Significantly

## Data Availability

Publicly available datasets were analyzed in this study. This data can be found here: https://opendata.pku.edu.cn/dataset.xhtml?persistentId=doi:10.18170/DVN/45LCSO (accessed on 20 July 2022).
